# A challenging diagnosis: Case report of oral metastasis from gastric adenocarcinoma mimicking pyogenic granuloma

**DOI:** 10.1097/MD.0000000000009934

**Published:** 2018-03-30

**Authors:** Ciro Dantas Soares, Breno Amaral Rocha, Lívia Máris Ribeiro Paranaiba, Mário Rodrigues de Melo-Filho, Jacks Jorge, Maria Goretti Freire de Carvalho, Oslei Paes de Almeida

**Affiliations:** a Piracicaba Dental School, University of Campinas, Piracicaba, São Paulo; b Oncologic Dentistry Service, Santa Casa Hospital, Montes Claros, Minas Gerais; c Dental School, University of Montes Claros, Montes Claros, Minas Gerais; d Health Science Program, State University of Montes Claros, Montes Claros, Minas Gerais; e Laboratório de Citopatologia, Private Service, Natal, Rio Grande do Norte, Brazil.

**Keywords:** gastric carcinoma, immunohistochemistry, metastasis, oral cavity

## Abstract

**Rationale:**

Oral metastases occur more commonly in bone, but can also manifest in soft tissues and eventually resemble a reactive lesion. Few cases of oral metastases mimicking reactive lesions in soft tissues have been reported to date.

**Patient concerns:**

We report a metastasis of gastric carcinoma (GC) to the oral mucosa without bone involvement in a 43 yom clinically and microscopically mimicking a reactive lesion. The patient related that the lesion had 1 month of evolution, and the ulcerated area suggested the lesion was related to trauma.

**Diagnoses:**

The histopathological examination of the lesion revealed an exuberant granulation tissue with few neoplastic cells, and the initial diagnosis of pyogenic granuloma was considered. In a second analysis, clusters of clear cells morphologically similar to degenerating mucous cells or macrophages, positive for Cytokeratin (CK)-20, and CDX2 were found. At the moment, it was confirmed the presence of a primary GC in the patient.

**Interventions:**

A palliative radiotherapy/chemotherapy was started.

**Outcomes:**

However, the patient died 3 months after the diagnosis of oral metastasis.

**Lessons:**

This report highlights the importance of careful clinical and microscopic examinations in cases of oral metastasis that may mimic a reactive lesion.

## 1 Introduction

Metastatic tumors of the mouth represent only 1% of all malignancies affecting this region. Usually oral metastases involve the jawbones and more rarely the soft tissues.^[1]^ These metastases can be challenging either clinically and microscopically for the correct diagnosis, and eventually can be mistaken for reactive lesions that are common in the mouth.^[2]^ It is also important to consider that approximately 25% of the oral metastases comprise the first evidence of an undiscovered malignancy at a distant site.^[3,4]^

Concerning the oral mucosa, the most common sites for metastasis are the gingiva, followed by the tongue and with less frequency the remaining oral soft tissues.^[3]^ Metastases in oral soft tissues usually manifest as ulcerated lesions or masses causing swellings. In the mouth, a few cases of metastases resembling pyogenic granuloma were reported, and it seems that this type of presentation is more common in the skin.^[5,6]^

The major primary sites presenting metastases to the mouth include lungs, kidney, liver, and prostate for men, and breast, uterus, ovaries, kidney, and colorectum for women.^[1,2]^ Oral metastases from gastric adenocarcinoma (GC) are rare, although this malignancy represents the fourth most common cancer in man and the second most frequent cause of human cancer death.^[7,8]^

In this report, we describe a metastatic oral mucosa lesion from gastric adenocarcinoma, clinically and microscopically resembling a pyogenic granuloma.

## 2 Case report

The authors read the Helsinki Declaration and followed its guidelines in this study.

**Figure 1:**
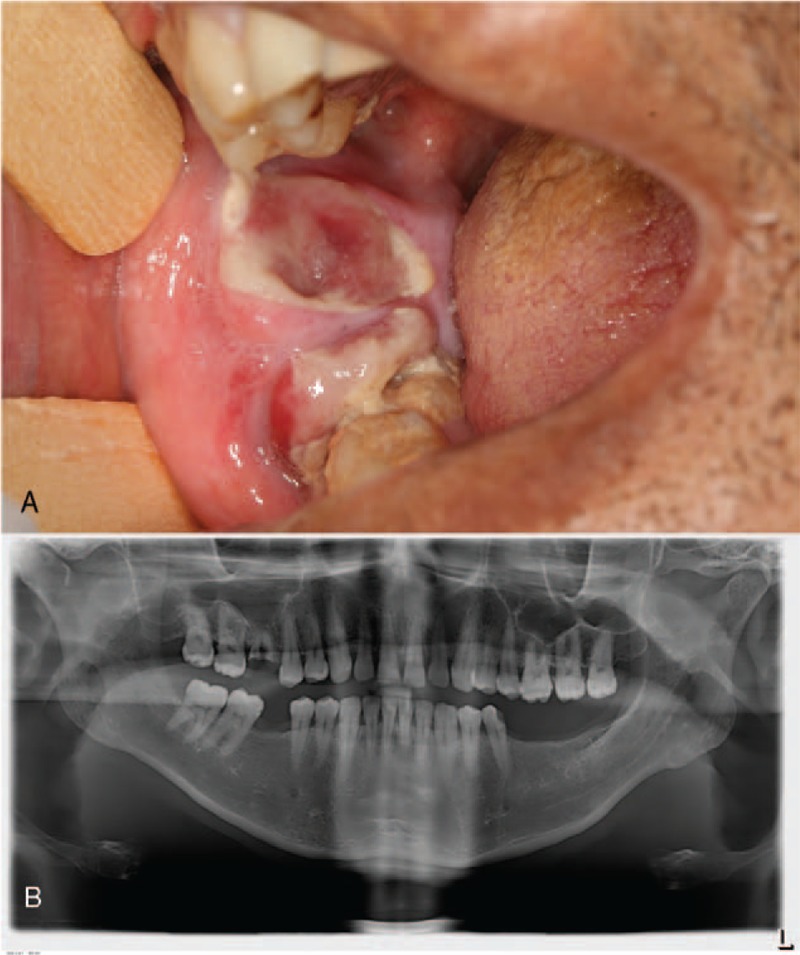
Clinical and radiographic appearances of metastatic gastric carcinoma into the mouth. (A) Intraoral mass involving the molar region of the right mandible. (B) Panoramic radiograph showing no bone involvement of the affected area.

Our service received a biopsy of a 43-year-old male for evaluation of an exophytic ulcerated mass involving the posterior region of the right mandible, with clinical hypothesis of a pyogenic granuloma or peripheral giant cell lesion. According to the patient, the lesion had 1 month of evolution, and the ulcerated area suggested the lesion was related to trauma (Fig. 1). A panoramic radiography revealed no alterations in the adjacent mandibular bone (Fig. 1).

**Figure 2:**
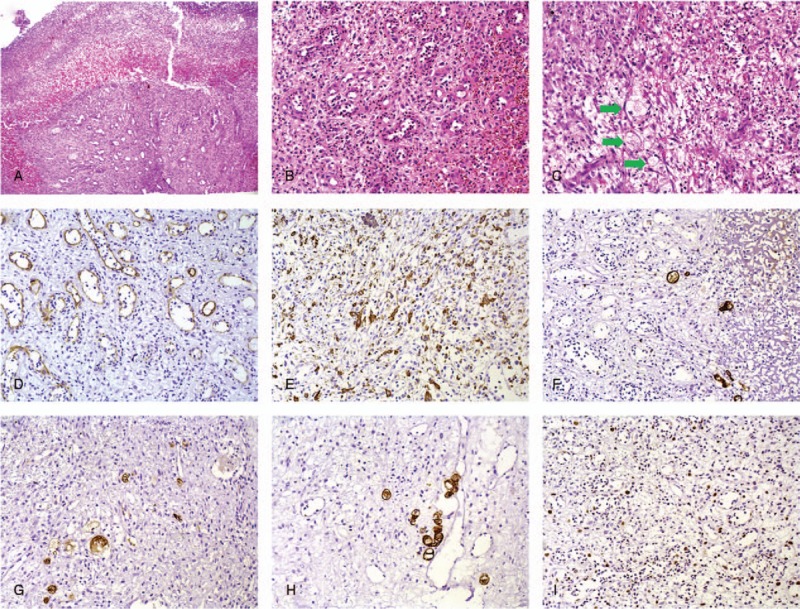
Microscopic findings of the first analysis. (A) Mucosa showing extensive ulceration covered by a fibrinopurulent membrane and subjacent exuberant granulation tissue. (B) Inflammatory infiltrate of lymphocytes and neutrophils and newly formed vessels, corresponding to pyogenic granuloma. (C) Few inconspicuous clear cells morphologically mimicking degenerated mucous cells or macrophages (green arrows), characterized by a large indistinct granular cytoplasm, small and pyknotic nuclei. Newly formed vessels were highlighted by the expression of CD34 (D), and numerous macrophages by CD68 (E), characterizing the granulation tissue. Clear cells were positive to pan-cytokeratin (AE1AE3) (F), CK -7 (G), CK -20 (H), and Ki67 (I).

An incisional biopsy was realized, and the histopathologic analysis disclosed an ulcerated lesion covered by a fibrinopurulent membrane, showing a predominance in the lamina propria of an exuberant granulation tissue (Fig. 2) formed by inflammatory cells, neovascularization, and few clear cells considered as degenerating mucous cells or macrophages (Fig. 2). Therefore, pyogenic granuloma was initially considered as the diagnosis, and it was suggested a most detailed analysis of the lesion.

**Figure 3:**
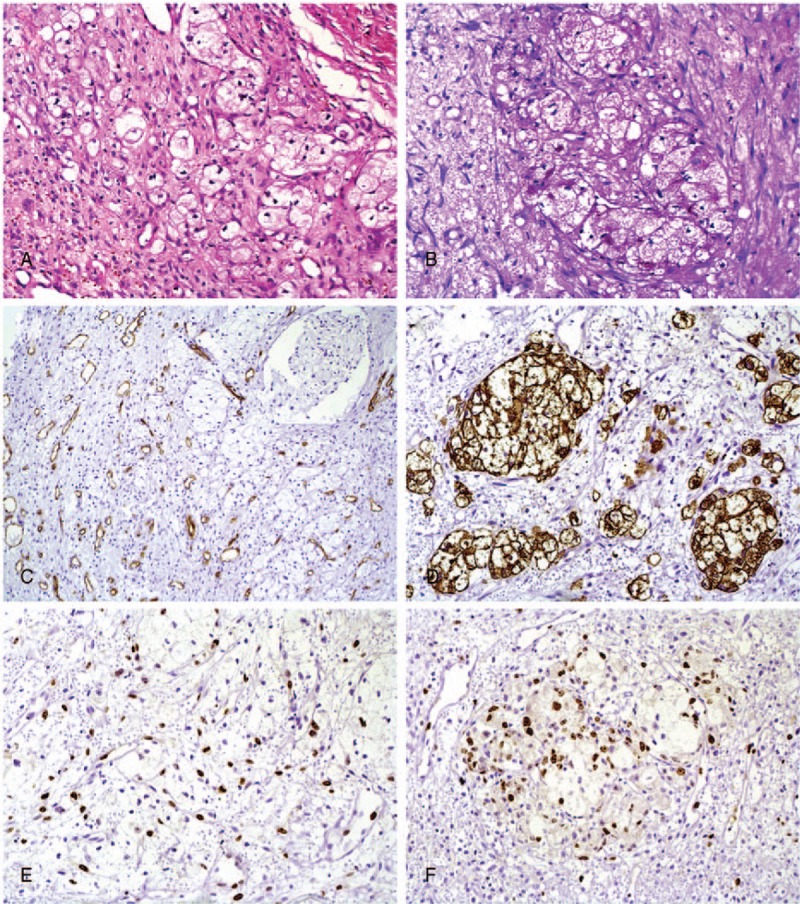
Histological and ìmmunohistochemical findings of the second analysis. (A) Clusters of clear cells dispersed in a granulation tissue. (B) Clear cells were positive for PAS with diastase. CD-34 to illustrate the presence of vascular vessels of the granulation tissue (C). Neoplastic clear cells demonstrated a high positivity for CK-20 (D), Ki67 (E), and CDX2 (F).

A second analysis revealed clusters of clear cells were evident, that by immunohistochemistry expressed cytokeratin (CK)-7, CK20, and CDX2, many positive for Ki-67, and negative to CD68 (Fig. 3). After a first analysis, similar results were found in the isolated clear cells. The final diagnosis was of a probable gastric metastasis. Clinical examination of the patient confirmed a primary adenocarcinoma of the stomach. A palliative radiotherapy/chemotherapy was started; however, the patient died 3 months after the diagnosis of oral metastasis

## 3 Discussion

GC is the fourth most common cancer in humans and the second cause of cancer death^[8]^; however, it uncommonly causes distant metastases, usually involving bones of the vertebrae, pelvis ribs, and skull,^[9,10]^ and rarely the gnathic bones. In this report, we describe a metastasis of a gastric adenocarcinoma into the buccal mucosa of a 43 yom that initially was considered as pyogenic granuloma. The lesion was ulcerated and exophytic involving the molar area of the right mandible. Histopathological examination of the biopsy disclosed an exuberant granulation tissue covered by fibrinopurulent membrane, with few clear cells considered as macrophages. A second analysis was performed, and small clusters of clear cells presenting a degenerative aspect were found. By immunohistochemistry, it was concluded that these clear cells represented metastasis of a GC. Isolated clear cells of the first analysis also proved to be neoplastic gastric cells. The diagnosis was clinically confirmed as the patient presented a primary adenocarcinoma of the stomach. This case illustrates that oral metastases may mimic reactive lesions both clinically and microscopically urging the necessity of careful examination. The first analysis displayed mainly granulation tissue, as the lesion was ulcerated due to dental trauma and this is common in many lesions of the mouth, indicating necessity of deeper biopsies. In fact, small and superficial biopsies are common for oral lesions, frequently difficulting the correct diagnosis.

Metastatic GC to the oral cavity tends to present signet ring cell,^[11,12]^ but glandular pattern and nests of epithelial gastric cells also have been reported.^[13–15]^ However, on the present case predominated an exuberant granulation tissue, suggesting a reactive lesion as pyogenic granuloma, and only on second analysis clusters of clear cells were more evident that represented metastasis of a gastric adenocarcinoma.

Metastatic GC to the oral cavity was previously reported including gingiva, soft palate, and soft tissues of the mandible.^[16]^ These lesions showed no bone involvement, as the present case, indicating a probable predilection of GC to metastasize to soft tissues of the oral cavity. Sauerborn et al^[12]^ have revised the cases of gastric metastatic carcinoma to oral soft tissues. In 18 cases reported, 10 occurred in the mandibular region, whereas 6 involved the maxilla, one each the tongue and soft palate. However, it was not reported that these lesions resembled benign reactive lesions. Furthermore, the histologic aspect of these reported cases was all compatible with adenocarcinomas, whereas in the present case only few cells were initially found immersed in a granulation tissue. To our knowledge, this is the first report of a metastatic GC to oral cavity mimicking a pyogenic granuloma.

The mechanisms of the metastatic process to the oral soft tissues are not fully understood. The ability of the primary tumor to stimulate angiogenesis and invade adjacent vessels has been considered crucial for spreading and distant colonization by the neoplastic cells, resulting in metastasis. However, this process comprises a complex biological cascade as the neoplastic cells acquire high ability for motility and invasion, survival, and proliferation.^[17]^ In the oral soft tissues, angiogenesis associated with chronic inflammation is very common, and this can facilitate the metastatic process,^[18]^ particularly the gingiva and adjacent tissues. Other studies correlated tooth extractions as a promoting factor in the metastatic process,^[19]^ supported by the same hypothesis of chronic inflammation caused by this procedure.

Overall, a bad prognosis is expected for metastatic neoplasms to the oral cavity, and death is common after a few months of the initial diagnosis. However, palliative treatment is important to improve life quality and pain relief.

In conclusion, oral metastasis of gastric adenocarcinoma is rare, and it may eventually mimic a reactive process, difficulting the diagnosis as illustrated in this report.

## Author contributions

**Conceptualization:** C.D. Soares, L.M.R. Paranaiba, M.G.F. Carvalho.

**Data curation:** C.D. Soares, M.G.F. Carvalho.

**Formal analysis:** M.G.F. Carvalho.

**Funding acquisition:** J. Jorge, O.P. Almeida.

**Investigation:** C.D. Soares, M.G.F. Carvalho.

**Methodology:** C.D. Soares, L.M.R. Paranaiba, M.G.F. Carvalho.

**Project administration:** J. Jorge, M.G.F. Carvalho, O.P. Almeida.

**Resources:** M.R. Melo-Filho.

**Supervision:** B.A. Rocha.

**Writing – original draft:** C.D. Soares, M.G.F. Carvalho.

**Writing – review & editing:** J. Jorge, M.G.F. Carvalho, O.P. Almeida.
